# Supplementary material for: A Critical View of the Application of the APEX Software (Aqueous Photochemistry of Environmentally-Occurring Xenobiotics) to Predict Photoreaction Kinetics in Surface Freshwaters
Source: Molecules. 2019 Dec 18;25(1):9. doi: 10.3390/molecules25010009 (PMC7017383; doi:10.3390/molecules25010009)
Supplement: Supplementary file 1 [file molecules-25-00009-s001.zip › 3.2.4_gcc-4.4.0.zip!/3.2.4_gcc-4.4.0/3.2.4_gcc-4.4.0/doc/octave/html/interpreter/Startup-Files.html]

Startup Files - Untitled


Previous: Command Line Options,
Up: Invoking Octave from the Command Line


---

#### 2.1.2 Startup Files

When Octave starts, it looks for commands to execute from the files in
the following list. These files may contain any valid Octave commands,
including function definitions.

octave-home`/share/octave/site/m/startup/octaverc`: where octave-home is the directory in which Octave is installed (the default is ). This file is provided so that changes to the default Octave environment can be made globally for all users at your site for all versions of Octave you have installed. Care should be taken when making changes to this file since all users of Octave at your site will be affected. The default file may be overridden by the environment variable `OCTAVE_SITE_INITFILE`. octave-home`/share/octave/`version`/m/startup/octaverc`: where octave-home is the directory in which Octave is installed (the default is ), and version is the version number of Octave. This file is provided so that changes to the default Octave environment can be made globally for all users of a particular version of Octave. Care should be taken when making changes to this file since all users of Octave at your site will be affected. The default file may be overridden by the environment variable `OCTAVE_VERSION_INITFILE`. `~/.octaverc`: This file is used to make personal changes to the default Octave environment. `.octaverc`: This file can be used to make changes to the default Octave environment for a particular project. Octave searches for this file in the current directory after it reads ~/.octaverc. Any use of the `cd` command in the ~/.octaverc file will affect the directory where Octave searches for .octaverc. If you start Octave in your home directory, commands from the file ~/.octaverc will only be executed once.

A message will be displayed as each of the startup files is read if you
invoke Octave with the `--verbose` option but without the
`--silent` option.
